# Comparative analysis of transcriptional profiles of *Schistosoma japonicum* adult worms derived from primary-infected and re-infected water buffaloes

**DOI:** 10.1186/s13071-019-3600-y

**Published:** 2019-07-11

**Authors:** Yudan Mao, Chuanchuan He, Hao Li, Ke Lu, Zhiqiang Fu, Yang Hong, Yamei Jin, Jiaojiao Lin, Xin Zhang, Jinming Liu

**Affiliations:** Shanghai Veterinary Research Institute, Chinese Academy of Agricultural Sciences, Key Laboratory of Animal Parasitology, Ministry of Agriculture, Shanghai, 200241 People’s Republic of China

**Keywords:** Comparative analysis, Primary-infected, Re-infected, RNA-Seq, *Schistosoma japonicum*, Water buffalo

## Abstract

**Background:**

*Schistosoma japonicum* (*S. japonicum*) is an important zoonotic parasite that is prevalent in China and parts of Southeast Asia. Water buffaloes are an important reservoir and the main transmission sources of *S. japonicum*. However, self-curing and resistance to re-infection have been observed in water buffaloes.

**Results:**

In this study, we compared the morphometry and differences in transcriptional expression of adult *S. japonicum* worms recovered from primary-infected and re-infected water buffaloes using Illumina RNA-sequencing (RNA-Seq) technology. Results of morphometry analysis revealed that adult *S. japonicum* worms recovered from re-infected water buffaloes were runtish with smaller organs. The ventral length of male worms was shorter in re-infected buffaloes (328 ± 13 *vs* 273 ± 8 µm, *P* < 0.05), and in female worms the oral sucker length (44 ± 3 *vs* 33 ± 5 µm, *P* < 0.05), ovary length (578 ± 23 *vs* 297 ± 27 µm, *P* < 0.05) and width (150 ± 8 *vs* 104 ± 9 µm, *P* < 0.05) were shorter, with fewer eggs in the uteri (41 ± 2 *vs* 12 ± 1, *P* < 0.05). Of 13,605 identified genes, 112 were differentially expressed, including 51 upregulated and 61 downregulated genes, in worms from re-infected compared with primary-infected water buffaloes. Gene ontology (GO) enrichment analysis revealed that GO terms such as “oxidation-reduction process”, “calcium-dependent phospholipid binding”, “lipid binding” and “calcium ion binding” were significantly enriched in downregulated genes, whereas GO terms related to metabolism and biosynthesis were significantly enriched in upregulated genes. The results revealed that the downregulation of some important genes might contribute to a reduction in worm numbers and maldevelopment of surviving worms in re-infected water buffaloes. Furthermore, upregulation of genes related to metabolic processes and biosynthesis might be a compensatory mechanism of worms in disadvantageous environments.

**Conclusions:**

To our knowledge, our results present the first large-scale transcriptional expression study identifying the differences between adult *S. japonicum* worms from primary-infected and re-infected water buffaloes, and particularly emphasize differential expression that may affect the survival and growth of worms in re-infected water buffalo. This will provide new insight into screening for anti-schistosome targets and vaccine candidates.

**Electronic supplementary material:**

The online version of this article (10.1186/s13071-019-3600-y) contains supplementary material, which is available to authorized users.

## Background

Schistosomiasis, caused by infections with *Schistosoma* species, is endemic in over 70 countries and territories located in tropical and subtropical regions and remains a major public health problem worldwide [1]. *Schistosoma japonicum* is a zoonotic parasite that is prevalent in China and parts of Southeast Asia [2]. It has been reported that over 40 species of wild and domestic animals can become infected with *S. japonicum* [3, 4]. Generally, praziquantel (PZQ) is the only effective chemotherapeutic drug against schistosomiasis. However, its utility is limited in areas with high re-infection and resistance rates. Additionally, low susceptibility of *Schistosoma mansoni* and *S. japonicum* to PZQ has been induced after mass drug administration programmes [5, 6]. Therefore, novel targets for drugs and vaccines to treat and eradicate schistosomiasis are urgently required.

Infection with *S. japonicum* causes serious economic losses to livestock farms. Many investigations have revealed that domestic animals, bovines in particular, are the major infectious source and play the most important role in the transmission of schistosomiasis in China [7, 8]. Thus, water buffaloes have been considered a target animal for schistosomiasis control in China for several decades [9]. A veterinary vaccine for domesticated bovines that blocks transmission would provide an effective approach to schistosomiasis control. Several research groups have focused on the development of efficient vaccines for buffaloes [10, 11]. However, the level of protection of these vaccines needs further improvement. Lack of knowledge about schistosome biology and host-parasite relationships in bovines remains an obstacle for vaccine development.

Recently, many public databases on the genomes, transcriptomes and proteomes of *Schistosoma* species, in particular *S. mansoni* and *S. japonicum* [12, 13], have been released, which has proved pivotal for the understanding of schistosome biology. In the past decade, a number of studies on gene expression and proteomic profiling of *Schistosoma* species have been performed using various analytical approaches. These studies mainly focus on expression patterns and features of sex-dependent, tissue-specific, host-associated and strain-specific genes [12, 14–16]. The findings have facilitated improved understanding of the molecular basis of schistosome developmental biology, host-parasite interactions and schistosomiasis pathogenesis. For example, studies from our research group have identified numerous differentially expressed genes (DEGs) that may influence parasite survival and development of schistosomula from susceptible BALB/c mice, less susceptible Wistar rats and resistant reed voles using comparative proteomic [17] and microarray analyses [18]. We have also examined gene expression profiles of *S. japonicum* worms derived from the natural hosts yellow cattle and water buffalo using comparative analysis of microarrays [19].

Water buffaloes are numerous and widely distributed in schistosomiasis-endemic regions and are a major reservoir for *Schistosoma* species in China [8]. Compared with other natural hosts, such as yellow cattle, goats and rabbits, water buffaloes are less susceptible to infection [20]. In addition, previous researchers reported self-curing in water buffaloes, as they observed decreases in infection rates with age [21] and age-related resistance to re-infection after PZQ treatment [22]. Recently, we observed a reduction of over 97% in the *S. japonicum* worm burden, shorter female worms, and a reduction of over 87.7% in egg counts in re-infected buffaloes [23]. Strong type-2 immune responses at the site of cercarial penetration have also been reported in challenged water buffaloes [24], which is significantly different from observations in other animal models such as mice. We also found that re-infected water buffaloes had significantly higher levels of interleukin (IL)-4, IL-10, interferon (IFN)-γ and specific immunoglobulin G (IgG) antibodies before challenge infection [23]. Here, we performed comparative analyses of gene expression profiles of *S. japonicum* adult worms from primary-infected and re-infected water buffaloes using deep RNA-transcriptome sequencing (RNA-Seq) technology. Our results will be of significance for understanding the mechanism of resistance and host-parasite relationships in re-infected water buffaloes and could provide a valuable resource for the identification of genes functionally related to parasite development in water buffaloes as well as the screening of anti-schistosome drug targets and vaccine candidates.

## Methods

### Infection protocol and parasite collection

**Fig. 1:**
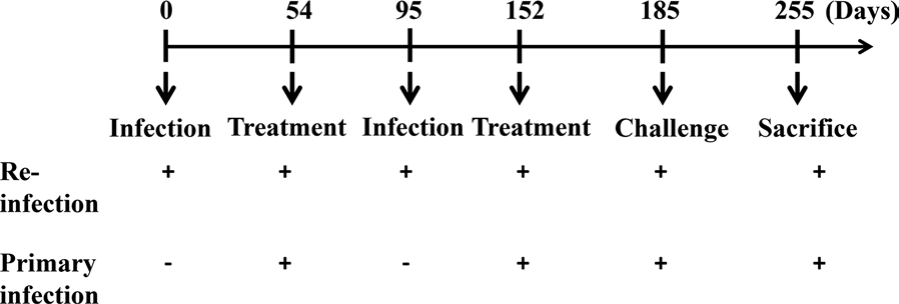
Schedule of animal infection, treatment and sacrifice. The primary infection and re-infection strategies for different groups were as shown. Briefly, re-infection group were infected with 3000 *S. japonicum* cercariae on day 0 and on day 95 and treated with PZQ on day 54 and day 152; primary infection group were not infected initially but were treated with PZQ at the same time points (on day 54 and day 152). On day 185, all animals were infected percutaneously with 3000 ± 100 cercariae. Animals were euthanized on day 255 (*n* = 3)

The parasites used in this study were collected from animals in the primary infection group (referred to in the previous study as the control group) and the re-infection group in the second trial of a study performed in 2017 to observe the degree of resistance of water buffaloes to re-infection with *S. japonicum*, as reported in our previous publication [23]. The infection protocol is summarized in Fig. 1 and briefly described as follows: six 15- to 18-month-old male water buffaloes (*Bubalus bubalis*) were purchased from non-schistosome-endemic areas in Nantong, Jiangsu Province, China and randomly divided into either the primary infection group or the re-infection group. *Schistosoma japonicum* cercariae used in all infections were shed from infected *Oncomelania hupensis* snails purchased from the Hunan Institute of Parasitic Diseases (Yueyang, China). Animals in the re-infection group were infected with 3000 *S. japonicum* cercariae on day 0 and on day 95 and treated with PZQ on day 54 and day 152. Water buffaloes in the primary infection group were not infected initially but were treated with PZQ at the same time points. In order to remove all *S. japonicum* worms in infected water buffaloes, PZQ was orally administered twice at 24 h intervals in each treatment at a dose of 25 mg/kg body weight up to a maximum of 10 g. On day 185, all animals were infected percutaneously with 3000 ± 100 cercariae. Animals were euthanized on day 255. Worms were obtained from three animals in the primary infection group and three animals in the re-infection group and designated C1, C2, C3, T1, T2 and T3, respectively. All parasites were manually washed twice with phosphate-buffered saline to remove residual host debris. Some worms were immediately preserved in RNAlater® (Invitrogen, Carlsbad, CA, USA) and stored in liquid nitrogen for subsequent RNA extraction. Five pairs of worms (five males and five females) from each animal were fixed with 10% neutral buffered formalin for morphological analysis.

### Morphological comparisons of worms collected from primary-infected and re-infected water buffaloes

A total of 30 pairs of worms, including 30 males and 30 females, were used for morphological comparisons. The paired worms were placed in ice water to separate the males and females. Slide specimens were prepared using the conventional method [25, 26] and observed under a microscope equipped with an automatic ACT-2U camera (Nikon, Tokyo, Japan). The total worm length, along with the dimensions of the oral sucker, ventral sucker and ovary were measured using NIS-Elements, a Nikon image analysis software. The number of eggs in the uterus of each female was counted. Data are expressed as the mean ± standard deviation. The results for each group were compared using Student’s t*-*tests in Excel 2013 (Microsoft Inc., Redmond, WA, USA) to examine intergroup differences. *P*-values < 0.05 were considered statistically significant.

### Total RNA isolation, qualification and transcriptomic library construction

Total RNA was extracted from individual worms collected from each water buffalo using TRIzol Reagent (Invitrogen), and contaminating genomic DNA was removed from RNA samples by treatment with RNase-free DNase I (New England Biolabs, Ipswich, MA, USA). RNA degradation and contamination were monitored on 1% agarose gels, a NanoPhotometer® spectrophotometer (IMPLEN, Westlake Village, CA, USA) and a Qubit® RNA Assay Kit with a Qubit^®^2.0 Fluorometer (Life Technologies, Carlsbad, CA, USA). Before RNA library construction, RNA integrity was assessed using an RNA Nano 6000 Assay Kit and Bioanalyzer 2100 system (Agilent Technologies, Santa Clara, CA, USA). RNA with an RNA integrity number (RIN) > 7.0 were considered of high enough quality for transcriptomic library construction and RNA sequencing according to the manufacturer’s instructions.

### Transcriptomic library preparation

For library construction, mRNA was purified from 3 μg of total RNA of each sample using poly-T oligo-attached magnetic beads (New England Biolabs) [27]. Transcriptome sequencing libraries were generated using an Illumina NEBNext® UltraTM RNA Library Prep Kit (New England Biolabs) according to the manufacturer’s instructions and index codes were added to attribute the sequences to the corresponding sample. Briefly, purified mRNA was cut into fragments and the cleaved mRNA fragments were reverse-transcribed into first-strand cDNA using random hexamers, followed by synthesis of double-strand cDNA. After blunting ends, the 250 to 300 bp fragments were purified using the AMPure XP system (Beckman Coulter, Brea, CA, USA). The purified cDNA fragments were then linked using an NEBNext Adaptor (New England Biolabs) with a hairpin loop structure and amplified by PCR. The AMPure XP system (Beckman Coulter) was used to purify the PCR products and the sample library quality was assessed using an Agilent Bioanalyzer 2100 system.

### Transcriptome sequencing and data analysis

Equal amounts of the 12 transcriptomic libraries were pooled and sequenced using an Illumina HiSeq™ 2000 (Illumina, San Diego, CA, USA) sequencing platform [28]. Raw reads in fastq format were processed using in-house perl scripts. These sequencing data are available at the NCBI Sequence Read Archive (SRA) database (www.ncbi.nlm.nih.gov/sra) under the accession number SRP168979. After calculating the Qpred ≤ 20 (Q20), Qpred ≤ 30 (Q30) and GC content of each sequence, clean data were obtained by removing the adapter from each sequence as well as any reads containing poly-N. Low quality reads were removed and the high quality clean reads were independently mapped twice to the genomic data (WormBase ParaSite, http://parasite.wormbase.org/Schistosoma_japonicum_prjea34885/Info/Index/) using Hisat2 v.2.0.5 [29]. Mapped reads for each sample were assembled using StringTie v.1.3.3b with a reference-based approach [30]. The expression level of each gene was estimated using the fragments per kilobase of transcript sequence per millions (FPKM) method. The DESeq2 R package v.1.16.1 was used for differential expression analysis of three biological replicates per condition. The Benjamini and Hochberg’s approach was applied to calculate *P*-values and expression fold change (FC), and genes with *P*-values < 0.05 and FC > 2 were considered differentially expressed. DEGs were functionally annotated using Blast2GO at http://www.blast2go.de [31] and assessed for enrichment using the clusterProfiler R package. The Kyoto Encyclopedia of Genes and Genomes (KEGG) database was used for DEG pathway annotation and enrichment analysis (http://www.genome.jp/kegg/).

### Verification of RNA sequencing data

The gene encoding *S. japonicum* NADH-ubiquinone reductase was employed as an endogenous reference gene [18]. The primer sequences used for qPCR were designed using Primer Express v.3.0 software (Applied Biosystems, Foster City, CA, USA) and are listed in Table 1. qPCR amplification was performed using a SYBR green kit (TaKaRa, Dalian, China) and an ABI 7500 Real-time System (Life Technologies). The qPCR cycling conditions were as follows: 95 °C for 3 min, followed by 40 cycles of 95 °C for 30 s, 56 °C for 30 s and 72 °C for 2 min. Melt curve analysis from 72 to 95 °C was performed to ensure a specific product was amplified in each reaction. The relative expression level of the genes was calculated using the 2^−ΔΔCT^ method [32].

**Table 1:** Primers used in quantitative PCR

Primer name	Sequence (5′–3′)	Product size (bp)
Upregulated genes		
Sjp_0125630F	CATGGATCTGTGCTCGCGTA	150
Sjp_0125630R	GCAAACCCGTGATTGGCAAG	
Novel03105F	GAGCAGACCGCTTCGATGTA	75
Novel03105R	CCGAATTTCGCTTGCCAGTC	
Novel00129F	ACTTCCCGCTCAAGATCACC	103
Novel00129R	ACTCGTAAGAGTGGGGTCCA	
Novel01404F	TTCTCAACAGCGTACACCCC	77
Novel01404R	GACCCAGTAGTGCAGACGTT	
Novel02684F	GGACGCCAGGTTAAGGAAGA	93
Novel02684R	TCTACCAGCCGAAGGAGTGT	
Downregulated genes		
Novel01481F	TTGGCCTCAATCGATCCTATCT	136
Novel01481R	CTGTGATTGCTTACCGTTTTGC	
Novel00143F	TGTGGGATTAAGGCCTACCA	95
Novel00143R	GCGAACGTAGAACAACTGCC	
Novel02333F	CGTTGTATGTGGATCATTTGTGC	70
Novel02333R	TTCATCTTTGCATTCACTACTACAC	
Sjp_0116000F	TGCAATTGTTCACCGTGATG	86
Sjp_0116000R	TGTCTCTGCAACATCTTGTGAT	
Novel00935F	CGCAATCAGATGTTCAGGGT	105
Novel00935R	TCGGAAACCCAATGTCTGGAT	

## Results

### Morphological differences between parasites from re-infected and primary-infected water buffaloes

Lengths of male and female worms were reported in our previous publication [23]. Here we analyzed other morphological characteristics and present the results from 30 pairs of worms (30 male and 30 female) in Table 2. Organs, including the oral sucker, ventral sucker and ovaries, of male and female worms from re-infected water buffaloes were smaller than those of worms from primary-infected animals. The differences were significant for the length of the ventral sucker in males (328 ± 13 *vs* 273 ± 8 µm; *t*_(14)_ = 26.63, *P* = 0.02), the length of the oral sucker in females (44 ± 3 *vs* 33 ± 5 µm; *t*_(14)_ = 8.52, *P* = 0.02) and the length (578 ± 23 *vs* 297 ± 27 µm; *t*_(14)_ = 40.31, *P* = 0.005) and width (150 ± 8 *vs* 104 ± 9 µm; *t*_(14)_ = 19.80, *P* = 0.02) of the ovary, but no other significant differences were found. We also observed that there were significantly fewer eggs in the uteri of female worms (41 ± 2 *vs* 12 ± 1; *t*_(14)_ = 56.16, *P* < 0.0001) from re-infected water buffaloes.

**Table 2:** Morphometry of *S. japonicum* worms recovered from primary-infected and re-infected water buffaloes

Characters	Primary infection	Re-infection
Male worms	(*n* = 15)	(*n* = 15)
Oral sucker length	261 ± 74	245 ± 23
Oral sucker width	176 ± 34	170±23
Ventral sucker length	328 ± 13	273 ± 8*
Ventral sucker width	236 ± 46	193 ± 24
Female worms	(*n* = 15)	(*n* = 15)
Oral sucker length	44 ± 3	33 ± 5*
Oral sucker width	34 ± 1	24 ± 7
Ventral sucker length	109 ± 8	94 ± 4
Ventral sucker width	87 ± 8	73 ± 3
Ovary length	578 ± 23	297 ± 27*
Ovary width	150 ± 8	104 ± 9*
No. of eggs in *utero*	41 ± 2	12 ± 1*

*Note*: Data are presented as the mean ± standard deviation; length and width in μm

* *P* < 0.05

### RNA extraction and RNA-Seq

The six RNA samples, including three from worms recovered from primary-infected water buffaloes and three from worms recovered from re-infected water buffaloes, had prominent *18S* and *28S* ribosomal peaks on agarose gels (data not shown). According to Bioanalyzer 2100 analysis, the RINs of all samples were > 7.0. These results showed that all RNA samples possessed high integrity and purity and could be used for further experiments.

**Fig. 2:**
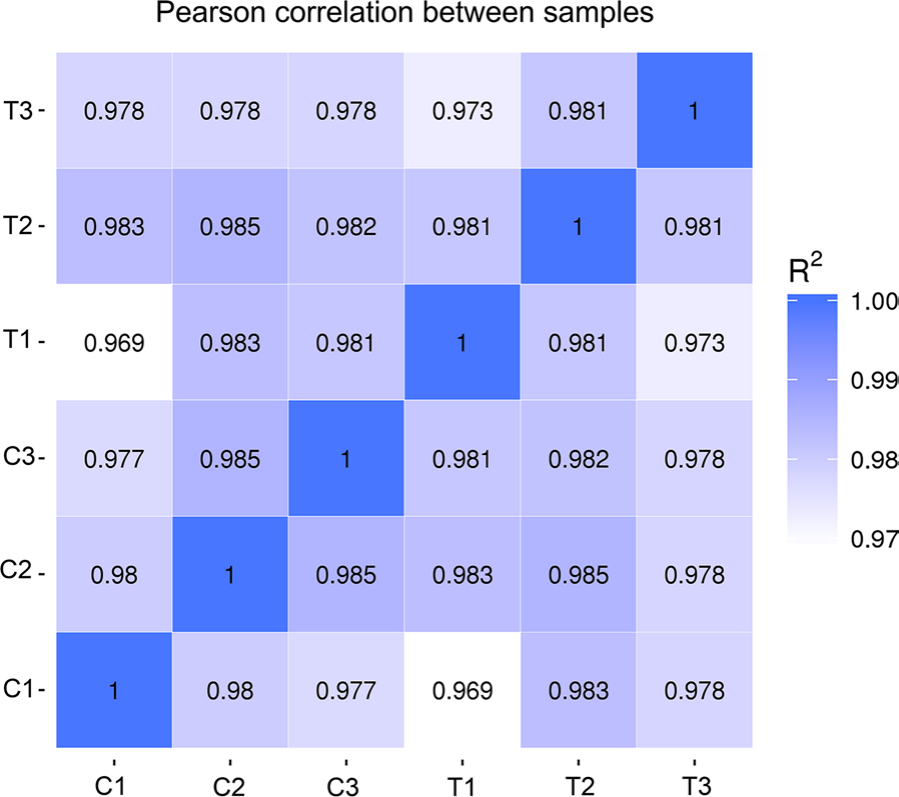
Heat map of correlation coefficients for all samples. C1, C2 and C3 represent samples from primary-infected water buffaloes. T1, T2 and T3 represent samples from re-infected water buffaloes. *R*^2^ represents the Pearsonʼs correlation coefficient

cDNA was generated and sequenced using an Illumina sequencing platform. Q30 was > 90% for each sample. After removing adaptors and low-quality reads, we obtained approximately 305,888,154 high quality reads in all samples. The average mapping rate of clean reads (51,832,023 reads per sample) to *S. japonicum* genomic data in two independent analyses was 79.545% (Table 3). The correlation coefficient of the transcriptional profiles between three biological replicates of the six samples was 0.969–0.985 (Fig. 2).

**Table 3:** Information on sequencing and mapping of reads to genomic data

Sample name	C1	C2	C3	T1	T2	T3
Total reads^a^	62,194,716	51,418,256	39,854,922	42,581,418	57,908,184	51,930,658
Total mapped (%)^b^	49,530,227 (79.64)	40,967,457 (79.67)	31,302,966 (78.54)	33,901,809 (79.62)	46,380,422 (80.09)	41,394,770 (79.71)
Multiple mapped (%)^c^	1,634,122 (2.63)	1,263,823 (2.46)	1,009,725 (2.53)	1,110,332 (2.61)	1,478,461 (2.55)	1,404,997 (2.71)
Uniquely mapped (%)^d^	47,896,105 (77.01)	39,703,634 (77.22)	30,293,241 (76.01)	32,791,477 (77.01)	44,901,961 (77.54)	39,989,773 (77.01)
Read-1 (%)	24,189,990 (38.89)	20,074,308 (39.04)	15,311,530 (38.42)	16,478,614 (38.7)	22,663,081 (39.14)	20,193,218 (38.88)
Read-2 (%)	23,706,115 (38.12)	19,629,326 (38.18)	14,981,711 (37.59)	16,312,863 (38.31)	22,238,880 (38.4)	19,796,555 (38.12)
Reads map to ‛+ʼ (%)^e^	23,840,204 (38.33)	19,786,847 (38.48)	15,100,441 (37.89)	16,352,368 (38.4)	22,390,777 (38.67)	19,940,268 (38.4)
Reads map to ‛-ʼ (%)^f^	24,055,901 (38.68)	19,916,787 (38.73)	15,192,800 (38.12)	16,439,109 (38.61)	22,511,184 (38.87)	20,049,505 (38.61)

^a^Sampling sequence filtered by sequencing data (clean data)

^b^Statistics on the number of sequencing sequences that can be mapped to the genome and the number in parentheses indicates the number of sequences (total mapped reads) as a percentage (%) of clean reads

^c^Quantitative statistics of sequencing sequences with multiple alignment positions on the reference sequence

^d^Quantitative statistics of sequencing sequences with unique alignment positions on the reference sequence

^e^The statistics of the sequencing sequence alignment to the positive strands on the genome

^f^The statistics of the sequencing sequences aligned to the negative strands on the genome

**Fig. 3:**
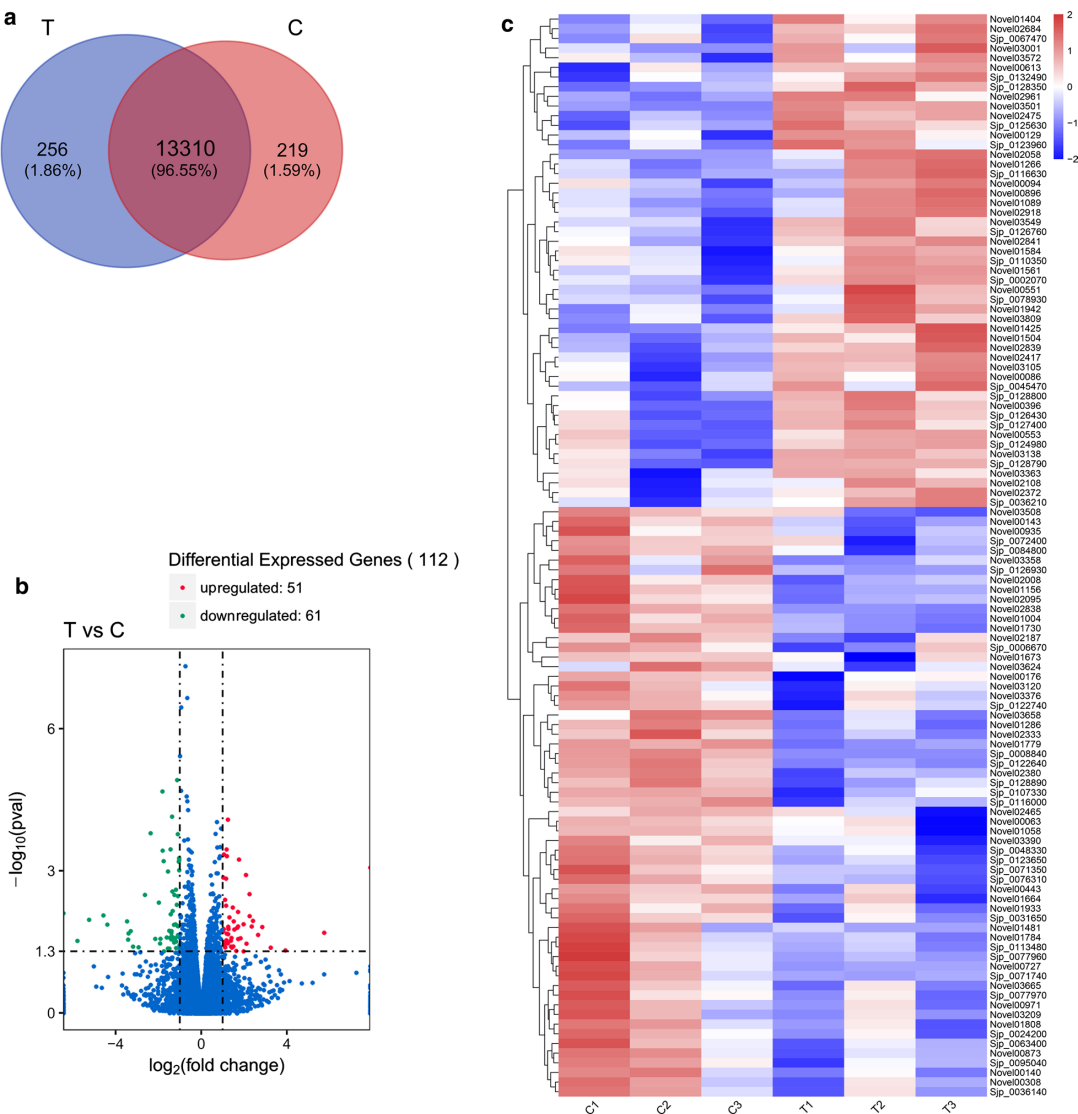
Transcription profile analysis of significantly differentially expressed genes. **a** Venn diagram showing the total number of expressed genes in each group in the large circles, and the number of genes expressed in both groups are shown in overlapping portion of the circles. **b** Volcano map showing significantly differentially expressed genes represented by red dots (upregulated) and green dots (downregulated). Genes that were not significantly differentially expressed are represented by blue dots. Abscissas indicate fold changes in gene expression between samples. Ordinates represent the statistical significance of the difference in gene expression. **c** Hierarchical clustering map of DEGs showing the overall FPKM. log10 (FPKM+1) values are scaled and clustered, with red representing upregulated genes and blue representing downregulated genes. Colors from red to blue indicate log10 (FPKM+1) values from largest to smallest. *Abbreviations*: C, worms from primary-infected water buffaloes; T, re-infected water buffaloes

We identified a total of 16,605 genes, including 3867 novel genes. Of these, 13,310 genes were expressed in adult worms recovered from both primary-infected and re-infected water buffaloes; 256 were only expressed in adult worms recovered from re-infected water buffaloes and 219 were only expressed in adult worms recovered from primary-infected water buffaloes (Fig. 3a). Using a *P*-value cutoff of < 0.05 and an FC cutoff of > 2, 112 genes were identified as DEGs (Fig. 3b). Compared with worms recovered from primary-infected water buffaloes, 51 genes were upregulated and 61 genes were downregulated in worms recovered from re-infected water buffaloes (Fig. 3b). DEGs that were successfully mapped to homologous proteins in the Gene DB database (http://www.genedb.org/Homepage) or annotated using the Swiss-Prot database (http://www.gpmaw.com/html/swiss-prot.html) are listed in Tables 4 and 5. Only genes that were successfully annotated using the Swiss-Prot database or found to have homologous proteins in the Gene DB database are listed. Cluster analysis for these DEGs in all six samples is shown in Fig. 3c.

**Table 4:** Upregulated genes in schistosomes recovered from re-infected water buffaloes normalized to primary-infected water buffaloes

Gene_ID	Protein homology	Annotation	Fold Change
Novel02058	Reverse transcriptase SjR1	na	na
Sjp_0123960	Immunogenic miracidial antigen 8I	na	15.20106
Sjp_0125630	Uncharacterized protein	Virion assembly	9.447286
Novel00553	Magnesium transporter nipa	Magnesium ion transmembrane transporter activity	6.277108
Novel01404	Protein TraX	Integral component of membrane	5.105315
Novel03501	Channel-forming transporter/TpsB family cytolysins activator	Protein transport	4.772681
Novel02684	Uncharacterized protein LOC106012791	Zinc ion binding	4.759467
Novel02475	Gag-Pol polyprotein	Threonine kinase	4.240105
Sjp_0110350	Asparagine-rich protein	na	3.560797
Sjp_0124980	SJCHGC04245 protein	Heterocyclic compound binding	3.293907
Sjp_0078930	Centrosomal protein of 162 kDa	Bacteriocin immunity, toxic substance binding	3.189433
Sjp_0132490	Retinol dehydrogenase 12	Catalytic activity	3.096417
Novel00396	Reverse transcriptase	Intrinsic component of membrane	2.91319
Novel02372	Polyprotein	DNA binding	2.854029
Sjp_0067470	Trehalose-6-phosphate hydrolase	Cation binding	2.620605
Novel03138	Ribonuclease III, putative	na	2.601241
Novel01504	SJCHGC02001 protein	Integral component of membrane	2.403939
Sjp_0002070	Malignant fibrous histiocytoma-amplified sequence 1 homolog	Purine nucleotide binding	2.379569
Sjp_0126430	ATP synthase subunit a	Purine nucleoside monophosphate biosynthetic process	2.360512
Sjp_0128790	Cytochrome *c* oxidase subunit 1	Oxidoreductase activity	2.287703
Sjp_0128350	Periodic tryptophan protein 2 homolog	na	2.270484
Novel02961	TPA: endonuclease-reverse transcriptase	Transferase activity	2.251209
Sjp_0127400	Cytochrome *c* oxidase subunits 1+2	Generation of precursor metabolites and energy, cytochrome-c oxidase activity	2.228696
Novel00551	Centrosomal protein of 162 kDa	Serine-type carboxypeptidase activity	2.226072
Sjp_0036210	Glycoprotein 3-alpha-l-fucosyltransferase A	Acting on L-amino acid peptides	2.111693
Sjp_0128800	ATP synthase subunit a	Purine nucleoside triphosphate biosynthetic	2.076854
Novel00613	Hypothetical protein MS3_03474	Porphobilinogen synthase activity	2.068952
Novel03809	Reverse transcriptase	RNA-directed DNA polymerase activity, nucleic acid binding	2.067948
Novel03549	TPA: endonuclease-reverse transcriptase	na	2.033408
Sjp_0126760	Glutamic acid-rich protein	Nucleobase-containing compound biosynthetic process	2.021043

*Notes*: Only genes that were successfully annotated using the Swiss-Prot database or found to have homologous proteins in the Gene DB database are listed

*Abbreviation*: na, not available

**Table 5:** Downregulated genes in schistosomes recovered from re-infected water buffaloes normalized to primary-infected water buffaloes

Gene_ID	Protein homology	Annotation	Fold change
Sjp_0008840	na	Integral component of membrane	na
Sjp_0126930	SJCHGC00271 protein	Transmembrane	0.0264
Novel00143	Uncharacterized protein	Mitochondrion	0.090296
Novel02333	Zinc finger and BTB domain-containing protein 16 (*Fundulus heteroclitus* (killifish; mummichog))	Integral component of membrane	0.09335
Sjp_0071350	Egg protein P3811	Transmembrane	0.09468
Novel00935	Egg protein CP11 (*Schistosoma japonicum*)	na	0.109659
Novel03624	Putative membrane protein	Transmembrane	0.131051
Novel00443	Transcriptional regulator	Regulation of transcription, DNA-templated	0.161175
Sjp_0048330	Beta-1,3-galactosyl-O-glycosyl-glycoprotein beta-1,6-N-acetylglucosaminyltransferase 3	Transferase activity	0.224891
Novel01156	Uncharacterized protein, SJCHGC08959 protein	Establishment of localization	0.262939
Sjp_0123650	CAP-Gly domain-containing linker protein 2, gene name:MS3_05295	Response to abiotic stimulus	0.266222
Sjp_0128890	Cytochrome *c* oxidase subunit 2	Aerobic respiration	0.295064
Novel02380	Gap-Pol polyprotein	na	0.297261
Novel02465	Reverse transcriptase	na	0.3384
Novel00176	SJCHGC04823 protein	na	0.339669
Sjp_0072400	Uncharacterized protein	Transmembrane	0.34707
Sjp_0031650	Probable delta-1-pyrroline-5-carboxylate synthase	Oxidoreductase activity	0.363745
Novel00063	Transposon Ty3-G Gag-Pol polyprotein	na	0.368695
Sjp_0113480	Annexin A3	Secretion by cell	0.371285
Sjp_0107330	Uncharacterized protein	Transmembrane	0.373661
Novel01784	Uncharacterized protein, HYPBUDRAFT_236782	Cytoskeleton	0.380482
Sjp_0095040	Amide-activated amiloride-sensitive sodium channel	Sodium ion transmembrane transporter activity	0.380508
Sjp_0084800	Beta-catenin-like protein 1	na	0.391179
Novel01664	Gag-Pol polyprotein	na	0.39513
Sjp_0122740	Annexin	na	0.414344
Novel03120	Hypothetical protein N336_01965, partial (*Phalacrocorax carbo*)	na	0.418181
Novel00971	Hypothetical protein Smp_144270 (*Schistosoma mansoni*)	na	0.431371
Novel01286	Uncharacterized protein	Transmembrane	0.434793
Sjp_0077960	Annexin A10	na	0.439215
Sjp_0122640	NADH-ubiquinone oxidoreductase chain 1	Inorganic cation transmembrane transporter activity	0.457011
Novel02095	Mitogen-activated protein kinase 15	na	0.467131
Sjp_0077970	Annexin A3	Phospholipase activity	0.468623
Novel01808	Hypothetical protein Smp_153120	na	0.472374
Novel02008	Reverse transcriptase	na	0.481698
Novel03665	Putative glycosyltransferase, partial	na	0.487137
Sjp_0071740	Uncharacterized protein	Transmembrane	0.487779
Novel03376	Hypothetical protein M959_01698, partial	Photosystem I reaction center	0.488354
Sjp_0076310	Tight junction protein ZO-1	Transcription factor TFIID complex	0.49107
Novel00873	Hypothetical protein MS3_01562	NADH dehydrogenase (ubiquinone) activity	0.496237

*Notes*: Only genes that were successfully annotated using the Swiss-Prot database or found to have homologous proteins in the Gene DB database are listed

*Abbreviation*: na, not available

### GO and KEGG enrichment analysis of differentially expressed genes

To obtain a better understanding of the enriched functions of DEGs, GO term enrichment analysis was performed. Of all identified genes, a total of 9012 (65.37%) were annotated (Additional file 1: Table S1). A total of 127 GO terms were enriched with *P* < 0.05 (Additional file 2: Table S2). Annotations of DEGs from the Swiss-Prot database are listed in Tables 4 and 5.

**Fig. 4:**
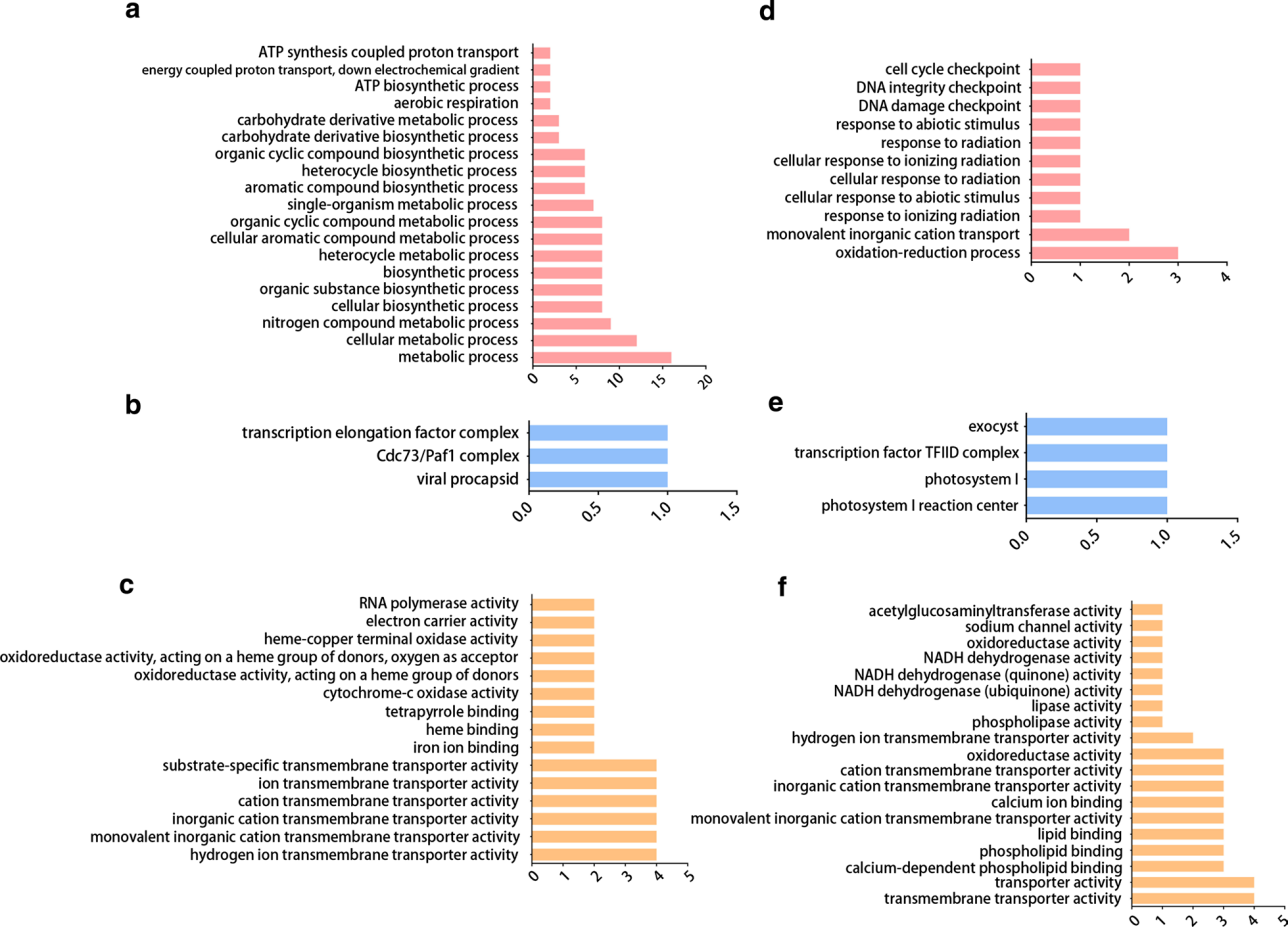
GO enrichment analysis of differentially expressed genes. **a**–**c** Upregulated genes. **d**–**f** Downregulated genes. GO terms were categorized as biological processes (**a**, **d**), cellular components (**b**, **e**) or molecular functions (**c**, **f**) using the Blast2Go program. Genes with no assigned GO terms were excluded from the graph. The ordinate shows the enriched GO term, and the abscissa shows the number of differentially expressed genes associated with the term

Of the upregulated genes in worms recovered from re-infected water buffaloes, 110 GO terms were significantly enriched with *P* < 0.05 (Fig. 4, Additional file 3: Table S3). Specifically, 83 GO terms, such as “metabolic process”, “cellular metabolic process”, “nitrogen compound metabolic process” and “cellular biosynthetic process”, were overrepresented in the “biological process” category; 24 GO terms, including “hydrogen ion transmembrane transporter activity”, “monovalent inorganic cation transmembrane transporter activity”, “inorganic cation transmembrane transporter activity” and “cation transmembrane transporter activity”, were overrepresented in the “molecular function” category; and three GO terms, including “viral procapsid”, “Cdc73/Paf1 complex” and “transcription elongation factor complex”, were overrepresented in the “cellular component” category.

Of the downregulated genes in worms recovered from re-infected water buffaloes, a total of 35 GO terms were significantly enriched with *P* < 0.05 (Fig. 4, Additional file 4: Table S4). In the “biological process” category, 11 GO terms, including “oxidation-reduction process”, “monovalent inorganic cation transport” and “response to ionizing radiation”, were significantly enriched; 19 GO terms, such as “transmembrane transporter activity”, “calcium-dependent phospholipid binding” and “phospholipid binding”, were significantly enriched in the “molecular function” category; and five GO terms, such as “photosystem I reaction center”, “transcription factor TFIID complex” and “exocyst”, were significantly enriched in the “cellular component” category.

Functional enrichment analyses of GO terms of DEGs were compared between the upregulated and downregulated genes in worms recovered from re-infected water buffaloes (Fig. 4, Table 6). Only DEGs that were successfully annotated using the Swiss-Prot database are listed. The analysis indicated that a large number of GO terms associated with metabolic processes and biosynthesis were enriched only in the upregulated genes. By contrast, the GO term oxidation-reduction was enriched only in downregulated genes. GO terms related to transportation and binding were significantly enriched in both the upregulated and downregulated genes. However, we found that upregulated and downregulated genes were associated with different binding functions. Upregulated genes were related to iron ion binding, heme binding and tetrapyrrole binding, whereas the downregulated genes were related to calcium-dependent phospholipid binding, lipid binding and calcium ion binding.

**Table 6:** Summary of GO enrichment analysis of differentially expressed genes

Function	Upregulated genes		Downregulated genes	
	Enrichment terms	Major genes	Enrichment terms	Major genes
Metabolism	GO:0008152, GO:0009404, GO:0071941, GO:0006807	Sjp_0126430, Novel00613, Sjp_0127400, Sjp_0067470, Sjp_0078930, Novel02961, Sjp_0036210, Novel02108, Novel02684, Sjp_0132490, Novel02475, Novel00129, Sjp_0128790, Sjp_0126760, Novel00551, Sjp_0128800, Novel0296	None	None
Transportation	GO:0015078, GO:0015077, GO:0022890, GO:0008324, GO:0015075 GO:0022891	Sjp_0127400, Sjp_0128800, Sjp_0128790, Sjp_0126430	GO:0022857, GO:0005215, GO:0015077, GO:0022890, GO:0008324, GO:0015078	Sjp_0095040, Sjp_0128890, Novel01156, Sjp_0122640
Biosynthesis	GO:0044249, GO:1901576, GO:0009058, GO:0019438, GO:0018130, GO:1901362, GO:0006754	Novel02684, Novel00551, Sjp_0128800, Sjp_0036210, Sjp_0126760, Novel02475, Sjp_0126430, Novel00613	None	None
Binding	GO:0005506, GO:0020037, GO:0046906, GO:0015643	Sjp_0128790, Sjp_0127400, Sjp_0078930	GO:0005544, GO:0005543, GO:0008289, GO:0005509	Sjp_0113480, Sjp_0077960, Sjp_0077970
Oxidation-reduction	None	None	GO:0055114	Sjp_0128890, Sjp_0031650, Sjp_0122640

*Note*: Only differentially expressed genes that were successfully annotated using the Swiss-Prot database are listed

**Fig. 5:**
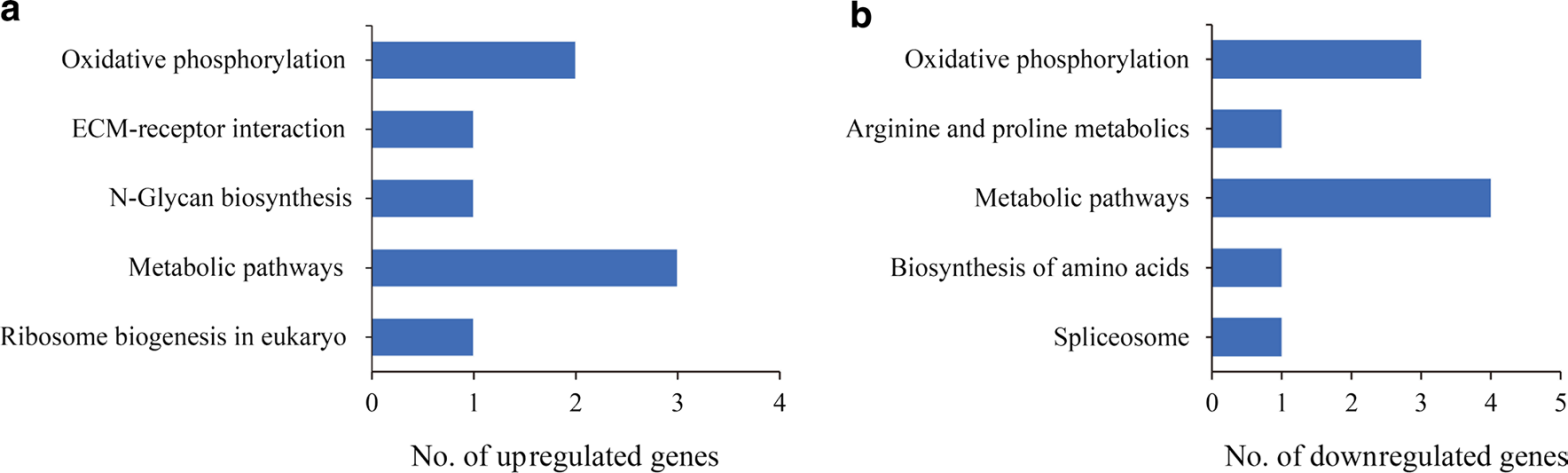
KEGG enrichment pathway analysis of differentially expressed genes. **a** Number of upregulated genes. **b** Number of downregulated genes

KEGG pathway enrichment analysis of DEGs indicated that both upregulated and downregulated genes were involved in oxidative phosphorylation and metabolic pathways. In addition, upregulated genes were also involved in extracellular matrix-receptor interactions, N-glycan biosynthesis and ribosome biogenesis in eukaryotes, and downregulated genes were associated with arginine and proline metabolism, biosynthesis of amino acids and spliceosome (Fig. 5, Additional file 5: Table S5).

### qPCR validation of RNA sequencing data

**Fig. 6:**
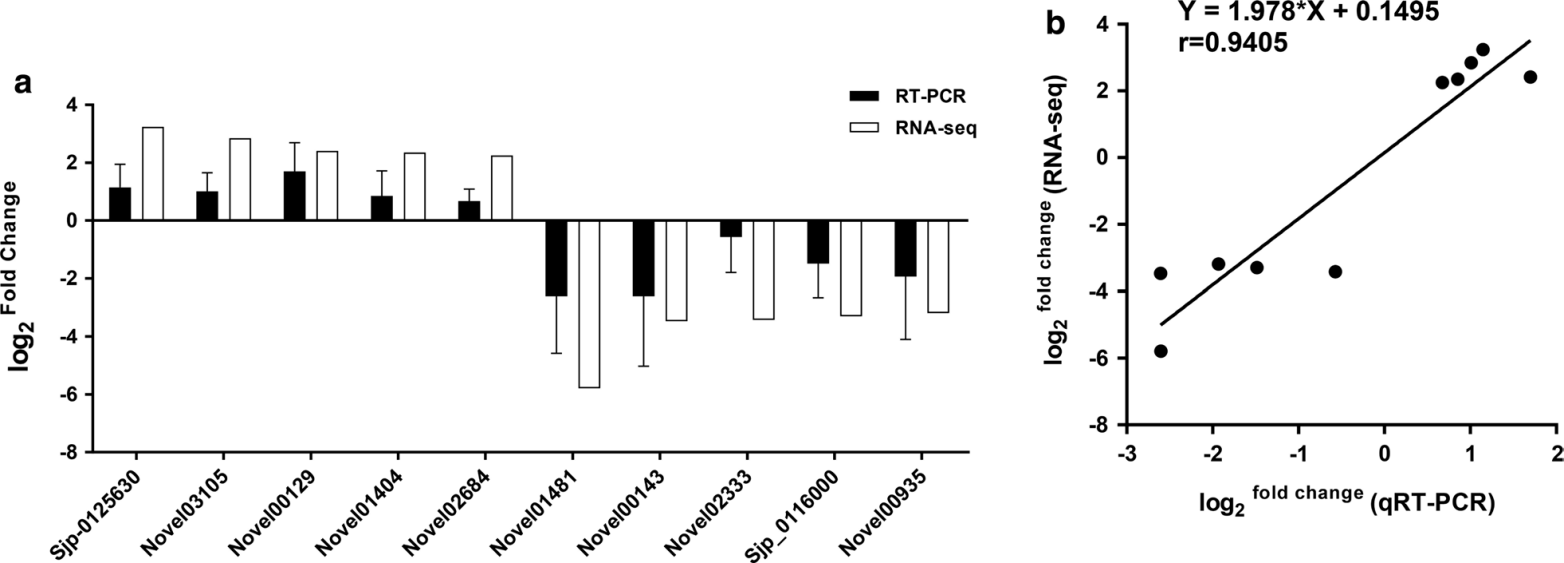
The expression of 10 selected genes with different expression patterns was quantified by qPCR analysis. **a** Ten selected genes with different expression patterns. **b** Correlation analysis of qPCR results and RNA-Seq data. Error bars represent the standard deviation for three technical replicates

To validate RNA-Seq data, qPCR was performed for 10 genes with different biological functions and expression patterns, including five upregulated genes and five downregulated genes, using NADPH as the housekeeping gene for internal normalization. The qPCR results corresponded with the RNA-Seq data (Fig. 6a), with a significant correlation coefficient of 0.9405 (*P* < 0.0001, *n* = 10) (Fig. 6b).

## Discussion

Intriguingly, water buffaloes are less susceptible to *S. japonicum* infection than yellow cattle, goats and rabbits [33] and self-curing has been observed in water buffaloes after infection [21]. In our previous publication, we reported a reduction in worm burdens of over 97.4% (*P* < 0.05) in re-infected compared to primary-infected water buffaloes [23]. Additionally, surviving adult female worms in water buffaloes previously exposed to *S. japonicum* were shorter in length. Here we further observed that organs including the oral sucker, ventral sucker and ovaries of both male and female worms were smaller in re-infected buffaloes than in primary-infected buffaloes, with a significant difference (*P* < 0.05) observed in the length of the ventral sucker of males, the length of the oral sucker of females and the length and width of the ovary. We also observed that there were significantly fewer eggs in the uteri of female worms of re-infected water buffaloes (*P* < 0.05). These results revealed that worm development was inhibited in water buffaloes previously exposed to *S. japonicum*. The susceptibility varies among diverse mammalian hosts to *S. japonicum* infection and the development of the worms in these hosts is different; for example, in susceptible hosts the development of the worm is favorable and in less susceptible hosts it is repressed [3, 20, 34, 35]. The growth environment of the worm in re-infected buffalo may be similar to that in an unsuitable host. Therefore, the worm development may be inhibited in the host environment.

Due to variations in the susceptibility of disparate mammalian hosts to *S. japonicum* infection, several recent comparative studies using proteomic analysis [36] and microarray analysis [19, 37] of *S. japonicum* from different hosts have been conducted, and numerous DEGs that may influence parasite survival and development have been identified. Yang et al. [19] compared the transcriptional profiles of adult schistosome worms recovered from yellow cattle and water buffaloes and found that several genes involved in transcription, transport, lipid metabolism, energy metabolism, nucleotides and energy and signaling pathways were differentially expressed in worms from these two hosts. In the present study, we compared the gene expression profiles of adult worms recovered from re-infected and primary-infected water buffaloes using RNA-Seq. We identified 112 DEGs in worms recovered from re-infected water buffaloes, including 61 upregulated genes and 51 downregulated genes. Genes related to transport, binding and oxidation-reduction were downregulated in adult worms from re-infected water buffaloes. For example, the GO terms “calcium-dependent phospholipid binding” (Sjp_0113480, Sjp_0077960, Sjp_0077970), “lipid binding” (Sjp_0113480, Sjp_0077960, Sjp_0077970) and “calcium ion binding” (Sjp_0077970, Sjp_0113480, Sjp_0077960) were enriched only in downregulated genes. *Schistosoma japonicum* parasites are unable to synthesize some key nutrient molecules such as fatty acids, sterols, purines and nine essential amino acids, including arginine and tyrosine [38]. Previous reports suggested that schistosomes cannot produce long-chain fatty acids and only obtain complex lipids for the synthesis of sterols and fatty acids from the host [39]. We hypothesize that the downregulation of these genes contributed to the reduction in worm numbers and the maldevelopment of surviving worms in re-infected water buffaloes by mechanisms other than acquired immunity [23]. Our functional enrichment analyses indicated that a large number of GO terms enriched in the upregulated genes were associated with metabolic processes and biosynthesis. In addition, we observed the overexpression of genes with a variety of binding-related activities, such as “zinc ion binding” (uncharacterized protein LOC106012791), “heterocyclic compound binding” (SJCHGC04245 protein), “DNA binding” (polyprotein), “cation binding” (trehalose-6-phosphate hydrolase), “purine nucleotide binding” (malignant fibrous histiocytoma-amplified sequence 1 homolog) and “nucleic acid binding” (reverse transcriptase). We speculated that the overexpression of genes related to metabolic processes and biosynthesis in surviving worms recovered from re-infected water buffaloes might be a compensatory mechanism by these worms to adapt to disadvantageous environments. Compensation mechanisms have been extensively studied in many diseases of humans and animals, such as Parkinson’s disease [40], facet joints arthritis [41] and unilateral kidney pathology[42]. In addition, synaptic plasticity deficit studies of Alzheimer’s disease have shown that the recruitment of nitric oxide (NO) serves a compensatory role to boost synaptic transmission and plasticity during early AD stages[43]. Presumably, in order to cope with the host’s unfavorable host environment, re-infected buffalo-derived worms employ a compensatory mechanism to integrate some molecules involved in metabolism and synthesis. Our results are consistent with the biology of *S. japonicum* and will help to clarify the basic molecular mechanisms underlying resistance to re-infection in water buffaloes, which might provide new insight into the genes functionally related to development as well as aid in screening of anti-schistosome drug targets and vaccine candidates.

Among the 112 DEGs, we observed that the transcript Sjp_0008840 was not expressed and that the transcript Novel02058 was expressed only in worms recovered from re-infected buffaloes. According to GO annotation analysis, transcript Sjp_0008840 is an integral membrane component. The transcript Novel02058 has 73.3% similarity at the gene level with reverse transcriptase SjR1 in *S. japonicum* [44] and, according to the UniProt database, functions in anaerobic aromatic compound degradation. We also found that the gene (Sjp_0078930) encoding toxic substance binding protein (centrosomal protein of 162 kDa) was upregulated in worms recovered from re-infected buffaloes. According to GO functional enrichment analysis, this centrosomal protein of 162 kDa is a critical molecule in centriole duplication during cell proliferation and development. Gudi et al. [45] showed that accurate centriole duplication is important for many cellular and physiological activities such as cell division and ciliogenesis. According to gene functional annotation analysis using the Gene DB database (http://www.genedb.org) [46], the gene is the DNA double-strand break repair protein RAD50 ATPase. DNA double-strand breaks are induced by environmental agents such as ionizing radiation and genotoxic chemicals and occurring spontaneously during DNA replication, threatening genomic stability [47]. DNA damage repair is fundamental to cell survival and cancer prevention [47, 48]. Thus, the effect of differential expression of these three genes on schistosome development is worthy of further investigation.

Reactive oxygen species (ROS) are naturally produced in cell compartments such as the peroxisome and mitochondria as well as at the plasma membrane and play key roles in signaling cell fate, growth and survival [49]. The observed increase in hydrogen peroxide (H_2_O_2_) detoxification capacity and resistance to multiple sources of ROS during *S. mansoni* development in the vertebrate host suggested that adult parasites are exposed to greater redox challenges than immature parasites [50, 51]. Similarly, we speculate that redox challenges in re-infected water buffaloes are greater than in primary-infected animals. Cytochrome c oxidase (CcO), which is a highly regulated enzyme, is believed to regulate mitochondrial oxidative metabolism and ATP synthesis [52, 53]. A previous study suggested that CcO dysfunction is associated with increased mitochondrial ROS production and cellular toxicity. Under normal physiological conditions, CcO is rate-limiting in the respiratory chain and its activity is an indicator of the oxidative capacity of cells [53]. Previous work indicated that the survival mechanisms of schistosomes in their definitive hosts included the production of protective antioxidant proteins, which neutralized oxidative damage caused by the host immune response as well as by worm-generated oxygen radicals [54, 55]. Oliveira et al. [49] found that *S. mansoni* employed a unique antioxidant network that is key to parasite survival and may be a valuable chemotherapeutic target. In the present study, three genes (Sjp_0128890, Sjp_0031650 and Sjp_0122640) related to oxidation-reduction processes were downregulated in worms recovered from re-infected water buffaloes, whereas the opposite expression pattern was observed for genes (Sjp_0127400 and Sjp_0128790) related to the redox pathway, including genes related to cytochrome c oxidase subunit 1 and cytochrome c oxidase subunit 1+2. These results indicate that redox reactions and ROS produced by circulating immune cells might play an essential role in parasite killing. Therefore, surviving worms have an increased ability to detoxify ROS and avoid redox imbalances and parasite cell death triggered by the host immune system.

Compared with cattle, fewer parasites are able to survive and mature in water buffaloes. The upregulation of elongase of very long-chain fatty acids (ELOVL) in adult schistosomes from water buffaloes might be a compensatory mechanism by parasites in less susceptible hosts to enable worm growth and development [56]. Here we found that genes related to metabolism and biosynthesis were upregulated in surviving worms in re-infected water buffaloes, which might be another compensatory mechanism by parasites in re-infected or immunized animals. Regarding metabolism, GO terms enriched in upregulated genes included metabolic processes, nitrogen compound metabolic processes, heterocycle metabolic processes, organic cyclic compound metabolic processes and carbohydrate derivative metabolic processes. Importantly, we found that three genes (Sjp_0067470, Sjp_0036210 and Sjp_0128800) were associated with most of these metabolic processes. For biosynthesis, GO terms enriched in upregulated genes included cellular biosynthetic processes, chemical component (i.e. organic substances, aromatic compounds, heterocycle and organic cyclic compounds) biosynthetic processes, energy substance (i.e. carbohydrate derivatives, ATP, purine nucleoside monophosphate, purine ribonucleoside monophosphate, nucleoside triphosphate and purine nucleoside triphosphate) biosynthetic processes and glycosyl compound biosynthetic processes. In particular, glycosyl compound biosynthetic processes are important for schistosome development, and schistosome glycoconjugates play an important role in the evasion mechanisms that worms utilize to evade host immunological responses [57, 58]. Regarding resistance to re-infection and self-curing in water buffaloes, it is generally accepted that these phenomenon are mainly due to specific immune responses produced by the animals in response to schistosome infection [59]. Studies have reported that some individuals develop partial resistance to re-infection after schistosomiasis infection and treatment and that high levels of isotype antibody IgE and low levels of IgG4 are closely related to soluble egg antigens (SEA) and adult worm antigen (SWA) in the resistant population [33, 60, 61]. We also found that a subset of transcripts involved in glycosylation, including beta-1, 3-galactosyl-O-glycosyl-glycoprotein beta-1, 6-N-acetylglucosaminyltransferase 3 (Sjp_0048330) and putative glycosyltransferase (Novel03665), were downregulated in worms recovered from re-infected water buffaloes. Glycosylation is a complicated biological process [62] and the global level of glycosylation and the functions of glycoconjugates in worms recovered from re-infected water buffaloes or other hosts that are resistant to re-infection require further study

Annexins belong to a multigene family of calcium-dependent phospholipid-binding proteins, many members of which have been identified in important eukaryotic phyla [63]. In humans, annexins interact closely with various cell membrane components to form networks on the cell surface that participate in the regulation of membrane organization, cell differentiation, migration and intracellular signaling by enzyme modulation and ion fluxes [64, 65]. Although annexins lack related signal peptides for secretion, many extracellular family members have been found to act as receptors for serum proteases on the endothelium and as inhibitors of neutrophil migration and blood coagulation [63]. In addition, some human annexin isoforms are involved in immunoregulatory functions such as the resolution of inflammation [66]. We identified four annexin domain-containing protein-encoding genes (Sjp_0113480, Sjp_0122740, Sjp_0077960 and Sjp_0077970) that were downregulated in worms recovered from re-infected hosts. This result indicates that the differential expression of these genes might affect the survival and development of schistosomes in their definitive host.

Information on the DEGs identified in the present study is essential to understanding the development of schistosomes in re-infected and primary-infected water buffaloes. In future studies, we will further investigate the *S. japonicum* genes identified in this study using *in vitro* culture or *in vivo* infection experiments using RNA interference (RNAi). We expect this to further contribute to our understanding of host-parasite relationships in re-infected and primary-infected water buffaloes and to provide helpful information for screening bovine schistosome vaccine candidates.

## Conclusions

In summary, we employed an Illumina RNA-Seq platform to identify DEGs in adult *S. japonicum* worms recovered from re-infected and primary-infected water buffaloes. We found that the downregulation of some important genes may have contributed to reductions in worm counts and the maldevelopment of surviving worms in re-infected water buffaloes. Furthermore, worms may have developed compensatory mechanisms to survive in the disadvantageous host environment. The identification of these DEGs provides new insights into mechanisms of resistance of water buffaloes to re-infection as well as useful information for comprehending the interactions between *S. japonicum* and its host.

## Additional files

**Additional file 1: Table S1.** Blast2GO annotation of all identified genes. Shown are genes from Blast2GO analysis.

**Additional file 2: Table S2.** Significantly enriched GO terms (*P* < 0.05).

**Additional file 3: Table S3.** Significantly enriched 110 GO terms (*P* < 0.05).

**Additional file 4: Table S4.** Significantly enriched 35 GO terms (*P* < 0.05).

**Additional file 5: Table S5.** KEGG pathway enrichment analysis of differently expressed genes.

## Data Availability

These sequencing data are available at the NCBI Sequence Read Archive (SRA) database at http://www.ncbi.nlm.nih.gov/sra under the Accession Number SRP168979.

## Abbreviations

RNA-Seq: RNA-sequencing
qPCR: quantitative PCR
GO: Gene Ontology
PZQ: praziquantel
DEGs: differentially expressed genes
IL: interleukin
IFN: interferon
IgG: immunoglobulin G
RIN: RNA integrity number
SRA: Sequence Read Archive
Q20: Qpred ≤ 20
Q30: Qpred ≤ 30
FPKM: fragments per kilobase of transcript sequence per millions
FC: fold change
KEGG: Kyoto Encyclopedia of Genes and Genomes
ROS: reactive oxygen species
CcO: cytochrome c oxidase
ELOVL: elongation of the very long chain fatty acids
SEA: soluble egg antigens
SWA: soluble adult worm antigen
RNAi: RNA interference
AAALAC: Association for Assessment and Accreditation of Laboratory Animal Care International
